# (*Z*)-6-Hy­droxy-1a,5-dimethyl-8-[(morpholin-4-yl)meth­yl]-2,3,6,7,7a,8,10a,10b-octa­hydro­oxireno[2′,3′:9,10]cyclo­deca­[1,2-*b*]furan-9(1a*H*)-one

**DOI:** 10.1107/S1600536811022616

**Published:** 2011-06-18

**Authors:** Mohamed Moumou, Ahmed Benharref, Moha Berraho, Lahcen El Ammari, Mohamed Akssira, Ahmed Elhakmaoui

**Affiliations:** a Laboratoire de Chimie des Substances Naturelles, URAC16 Faculté des Sciences Semlalia, BP 2390 Bd My Abdellah, 40000 Marrakech, Morocco; b Laboratoire de Chimie du Solide Appliqueé, Faculté des Sciences, Avenue Ibn, Battouta BP 1014 Rabat, Morocco; c Laboratoire de Chimie Bioorganique et Analytique, URAC 22, BP 146, FSTM, Université Hassan II, Mohammedia-Casablanca 20810 Mohammedia, Morocco

## Abstract

The title compound, C_19_H_29_NO_5_, was synthesized from 9α-hy­droxy­parthenolide (9α-hy­droxy-4,8-dimethyl-12-methylen-3,14-dioxatricyclo­[9.3.0.0^2^,^4^]tetra­dec-7-en-13-one), which was isolated from the chloro­form extract of the aerial parts of *Anvillea radiata*. The mol­ecule is built up from two fused five- and ten-membered rings with the (morpholin-4-yl)methyl group as a substituent. The five-membered lactone ring has an envelope conformation, whereas the ten-membered and the morpholine rings display approximate chair–chair and chair conformations, respectively. The dihedral angle between the ten-membered ring and the lactone ring is 27.93 (6)°. The crystal structure is stabilized by weak inter­molecular C—H⋯O hydrogen-bond inter­actions. An intra­molecular O—H⋯N hydrogen bond also occurs.

## Related literature

For background to the medicinal uses of the plant *Anvillea radiata*, see: Abdel Sattar *et al.* (1996 ▶); Bellakhdar (1997 ▶); El Hassany *et al.* (2004 ▶); Qureshi *et al.* (1990 ▶). For the reactivity of this sesquiterpene see: Der-Ren *et al.* (2006 ▶). For ring puckering parameters, see: Cremer & Pople (1975 ▶).
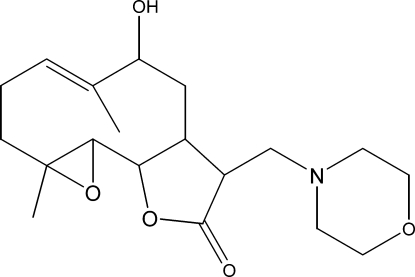


## Experimental

### 

#### Crystal data

C_19_H_29_NO_5_
*M* *_r_* = 351.43
Monoclinic, 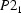

*a* = 11.7539 (3) Å
*b* = 6.8304 (2) Å
*c* = 11.8585 (3) Å
β = 101.328 (2)°
*V* = 933.50 (4) Å^3^
*Z* = 2
Mo *K*α radiation
μ = 0.09 mm^−1^
*T* = 298 K
0.45 × 0.33 × 0.12 mm

#### Data collection

Bruker APEXII CCD area-detector diffractometer
11514 measured reflections
2086 independent reflections
1987 reflections with *I* > 2σ(*I*)
*R*_int_ = 0.022

#### Refinement

*R*[*F* ^2^ > 2σ(*F* ^2^)] = 0.033
*wR*(*F* ^2^) = 0.095
*S* = 1.07
2086 reflections
229 parameters
1 restraint
H-atom parameters constrained
Δρ_max_ = 0.17 e Å^−3^
Δρ_min_ = −0.13 e Å^−3^

### 

Data collection: *APEX2* (Bruker, 2005 ▶); cell refinement: *APEX2* and *SAINT* (Bruker, 2005 ▶); data reduction: *SAINT*; program(s) used to solve structure: *SHELXS97* (Sheldrick, 2008 ▶); program(s) used to refine structure: *SHELXL97* (Sheldrick, 2008 ▶); molecular graphics: *ORTEP-3 for Windows* (Farrugia, 1997 ▶) and *PLATON* (Spek, 2009 ▶); software used to prepare material for publication: *WinGX* (Farrugia, 1999 ▶).

## Supplementary Material

Crystal structure: contains datablock(s) I, global. DOI: 10.1107/S1600536811022616/zl2378sup1.cif

Structure factors: contains datablock(s) I. DOI: 10.1107/S1600536811022616/zl2378Isup2.hkl

Additional supplementary materials:  crystallographic information; 3D view; checkCIF report

## Figures and Tables

**Table 1:** Hydrogen-bond geometry (Å, °)

*D*—H⋯*A*	*D*—H	H⋯*A*	*D*⋯*A*	*D*—H⋯*A*
O4—H4*A*⋯N	0.82	2.24	3.051 (2)	172
C2—H2*B*⋯O2^i^	0.97	2.51	3.324 (3)	142
C10—H10⋯O1^ii^	0.98	2.47	3.270 (2)	138

Symmetry codes: (i) 
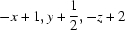
; (ii) 
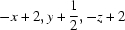
.

**Fig. 1.**
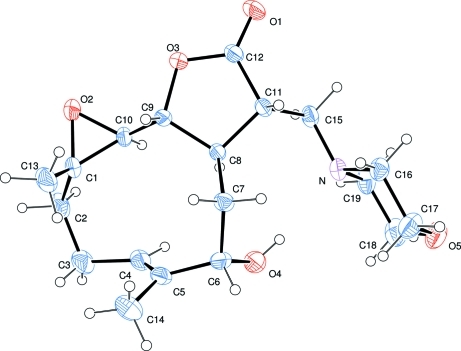
: Molecular structure of the title compound with the atom-labelling scheme. Displacement ellipsoids are drawn at the 30% probability level. H atoms are represented as small spheres of arbitrary radii.

**Fig. 2.**
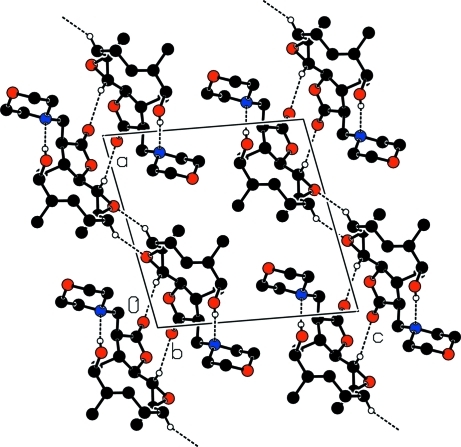
: Packing view showing the C–H···O and O–H···N hydrogen bonds as dashed lines. H atoms not involved in hydrogen bonding have been omitted for clarity.

## supplementary crystallographic information

### Comment

*Anvillea radiata* is a plant that grows in northern Africa and particularly in the two Maghreb countries, Morocco and Algeria. This plant is used in the traditional local medicine for the treatment of dysentery, gastric-intestinal disorders (Bellakhdar, 1997), and hypoglycemic activity (Qureshi *et al.*, 1990), and has been reported to have antitumor activity (Abdel Sattar *et al.*, 1996). In our study of different Moroccan endemic plants, we have demonstrated that the aerial parts of *Anvillea radiata* could be used as a renewable source of 9-hydroxyparthenolide (El Hassany *et al.*, 2004). In order to prepare products with a high added value that can be used in the pharmacology and cosmetics industry, we studied the chemical reactivity of this major constituent of *Anvillea radiata*. Thus, treatment of this sesquiterpene with an equivalent amount of morpholine in ethanol (Der-Ren *et al.*, 2006) led to (*Z*)-6-hydroxy-1a,5-dimethyl-8-(morpholinomethyl)-2,3,6,7,7a,8,10*a*,10*b*- octahydrooxireno[2',3': 9,10]cyclodeca[1,2-*b*]furan-9(1aH)-one, in a yield of 90%. The structure of this new product was determined by ^1^H and ^13^C NMR spectral analysis, IR and mass spectrometry, and was confirmed by its single-crystal X-ray structure. The molecule contains two fused rings which exhibit different conformations with a morpholin ring as a substituent to the lactone ring. The molecular structure of (I), Fig.1, shows the lactone ring to adopt an envelope conformation, as indicated by Cremer & Pople (1975) puckering parameters Q = 0.2O83(14) Å and φ = 68.2 (4)°. The ten-membered ring displays an approximate chair-chair conformation, while the morpholin ring has a perfect chair conformation with QT = 0.5690 (19) Å, θ2 = 0.00 (19)°, φ2 = 135 (6)°. In the crystal structure, the molecules are linked by C—H···O intermolecular hydrogen bonds into zigzag chains along the *a* axis (Fig.2). In addition an intramolecular O—H···N hydrogen bond is also observed.

### Experimental

A mixture of 9α-hydroxyparthenolide (0.5 g, 2 mmol) and one equivalent of morpholine in EtOH (20 ml) was stirred for one night at room temperature. The next day the reaction was stopped by adding water (10 ml) and extracted three times with ethyl acetate (3 *x* 20 ml). The combined organic layers were dried over anhydrous MgSO_4_, filtered and concentrated under vacuum to give 600 mg wite solid (1.8 mmol) which was recrystallized in ethyle acetate. Mp = 474–475 K (ethyl acetate); ^1^H NMR (300 MHz, CDCl_3_) δ 1.30 (H-13, s, 3H); 1.70 (H-14, s, 3H); 2.55 (H-15, m, 2H); 2.68 (H-16, H-19, t, *J* = 4.5 Hz, 4H); 3.10 (H-10, d, *J* = 8.70 Hz, 1H); 3.68 (H-17 H-18, t, *J* = 4.5 Hz, 4H); 3.95 (H-6, dd, *J_1_* = 1.2 Hz and *J_2_* = 11.5 Hz, 1H); 4.55 (H-9, dd, *J_1_* = 8,7 Hz, and *J_2_* = 9.3 Hz, 1H); 5.55 (H-4, dd, *J_1_* = 2,4 and *J_2_* = 12, 0 Hz, 1H); ^13^C RMN (300 MHZ, CDCl_3_)δ 16.83 (C-13); 17.13 (C-14); 23.04 (C-3); 36.65 (C-2); 37.09 (C-7); 37.83 (C-8); 44.27 (C-11); 54.03 (C-16, C-19); 59.95 (C-15); 60.86 (C-10); 66.17 (C-1); 67.69 (C-17, C-18); 70.94 (C-6); 82.87 (C-9); 120.97 (C-4); 137.37 (C-5); 177.50( C-12); IR (KBr): 3433 cm^-1^ (OH), 1766 cm^-1^ (lactone carbonyl), 1668 cm^-1^ (double bond); MS (EI, 70 eV): 351(*M*^+^).

### Refinement

All H atoms were fixed geometrically and treated as riding with C—H = 0.96 Å (methyl), 0.97 Å (methylene), 0. 98Å (methine) with *U*_iso_(H) = 1.2*U*_eq_ (methylene, methine) or *U*_iso_(H) = 1.5*U*_eq_ (methyl, OH). In the absence of significant anomalous scattering, the absolute configuration could not be reliably determined and thus 1606 Friedel pairs were merged and any references to the Flack parameter were removed.

### Crystal data

**Table:**

C_19_H_29_NO_5_	*F*(000) = 380
*M**_r_* = 351.43	*D*_x_ = 1.250 Mg m^−^^3^
Monoclinic, *P*2_1_	Mo *K*α radiation, λ = 0.71073 Å
Hall symbol: P 2yb	Cell parameters from 11515 reflections
*a* = 11.7539 (3) Å	θ = 1.8–26.4°
*b* = 6.8304 (2) Å	µ = 0.09 mm^−^^1^
*c* = 11.8585 (3) Å	*T* = 298 K
β = 101.328 (2)°	Prism, colourless
*V* = 933.50 (4) Å^3^	0.45 × 0.33 × 0.12 mm
*Z* = 2	

### Data collection

**Table:**

Bruker APEXII CCD area-detector diffractometer	1987 reflections with *I* > 2σ(*I*)
Radiation source: fine-focus sealed tube	*R*_int_ = 0.022
graphite	θ_max_ = 26.4°, θ_min_ = 1.8°
φ and ω scans	*h* = −14→14
11514 measured reflections	*k* = −7→8
2086 independent reflections	*l* = −14→14

### Refinement

**Table:**

Refinement on *F*^2^	Primary atom site location: structure-invariant direct methods
Least-squares matrix: full	Secondary atom site location: difference Fourier map
*R*[*F*^2^ > 2σ(*F*^2^)] = 0.033	Hydrogen site location: inferred from neighbouring sites
*wR*(*F*^2^) = 0.095	H-atom parameters constrained
*S* = 1.07	*w* = 1/[σ^2^(*F*_o_^2^) + (0.064*P*)^2^ + 0.0786*P*] where *P* = (*F*_o_^2^ + 2*F*_c_^2^)/3
2086 reflections	(Δ/σ)_max_ < 0.001
229 parameters	Δρ_max_ = 0.17 e Å^−^^3^
1 restraint	Δρ_min_ = −0.13 e Å^−^^3^

### Special details

**Table:**

Geometry. All s.u.'s (except the s.u. in the dihedral angle between two l.s. planes) are estimated using the full covariance matrix. The cell s.u.'s are taken into account individually in the estimation of s.u.'s in distances, angles and torsion angles; correlations between s.u.'s in cell parameters are only used when they are defined by crystal symmetry. An approximate (isotropic) treatment of cell s.u.'s is used for estimating s.u.'s involving l.s. planes.

### Fractional atomic coordinates and isotropic or equivalent isotropic displacement parameters (Å^2^)

**Table:**

	*x*	*y*	*z*	*U*_iso_*/*U*_eq_	
C4	0.68147 (17)	0.6737 (3)	0.7681 (2)	0.0506 (5)	
H4	0.7514	0.6984	0.8181	0.061*	
C1	0.59711 (14)	0.3364 (3)	0.86650 (18)	0.0446 (4)	
C2	0.56720 (18)	0.5347 (4)	0.9071 (2)	0.0602 (6)	
H2A	0.6204	0.5665	0.9782	0.072*	
H2B	0.4894	0.5311	0.9232	0.072*	
C3	0.5735 (2)	0.6950 (4)	0.8181 (3)	0.0662 (7)	
H3A	0.5056	0.6872	0.7569	0.079*	
H3B	0.5736	0.8224	0.8543	0.079*	
C5	0.68894 (16)	0.6245 (3)	0.66217 (19)	0.0490 (5)	
C6	0.80378 (17)	0.5685 (3)	0.63035 (17)	0.0456 (5)	
H6	0.7974	0.5963	0.5483	0.055*	
C7	0.82776 (15)	0.3483 (3)	0.64773 (14)	0.0379 (4)	
H7A	0.8859	0.3110	0.6039	0.045*	
H7B	0.7572	0.2775	0.6160	0.045*	
C8	0.86965 (12)	0.2824 (3)	0.77319 (13)	0.0298 (3)	
H8	0.8879	0.4000	0.8206	0.036*	
C11	0.97917 (13)	0.1537 (3)	0.79115 (13)	0.0339 (4)	
H11	0.9816	0.0817	0.7202	0.041*	
C12	0.96289 (15)	0.0110 (3)	0.88422 (15)	0.0392 (4)	
C9	0.78383 (13)	0.1581 (3)	0.82571 (13)	0.0320 (3)	
H9	0.7295	0.0892	0.7654	0.038*	
C10	0.71981 (13)	0.2807 (3)	0.89708 (14)	0.0367 (4)	
H10	0.7686	0.3785	0.9441	0.044*	
C14	0.5874 (2)	0.6021 (6)	0.5617 (2)	0.0801 (9)	
H14A	0.5162	0.6256	0.5876	0.120*	
H14B	0.5870	0.4718	0.5313	0.120*	
H14C	0.5950	0.6949	0.5028	0.120*	
C13	0.51592 (17)	0.2541 (4)	0.7628 (2)	0.0627 (6)	
H13A	0.5454	0.1310	0.7420	0.094*	
H13B	0.5101	0.3440	0.6997	0.094*	
H13C	0.4405	0.2349	0.7808	0.094*	
C15	1.09185 (13)	0.2656 (3)	0.82863 (14)	0.0408 (4)	
H15A	1.0922	0.3251	0.9030	0.049*	
H15B	1.1562	0.1742	0.8378	0.049*	
C16	1.14927 (17)	0.3355 (3)	0.64750 (16)	0.0451 (4)	
H16A	1.0909	0.2467	0.6069	0.054*	
H16B	1.2201	0.2617	0.6734	0.054*	
C17	1.1711 (2)	0.4972 (4)	0.5678 (2)	0.0615 (6)	
H17A	1.1966	0.4405	0.5019	0.074*	
H17B	1.0992	0.5669	0.5398	0.074*	
C19	1.19698 (17)	0.5597 (4)	0.80346 (19)	0.0526 (5)	
H19A	1.2686	0.4910	0.8339	0.063*	
H19B	1.1701	0.6210	0.8672	0.063*	
C18	1.2193 (2)	0.7138 (4)	0.7208 (3)	0.0680 (7)	
H18A	1.1490	0.7893	0.6953	0.082*	
H18B	1.2788	0.8022	0.7597	0.082*	
N	1.10962 (11)	0.4195 (3)	0.74672 (12)	0.0387 (4)	
O1	1.03350 (12)	−0.0969 (3)	0.93835 (13)	0.0577 (4)	
O2	0.63581 (11)	0.1901 (3)	0.95455 (13)	0.0552 (4)	
O3	0.85411 (10)	0.0191 (2)	0.90318 (10)	0.0397 (3)	
O4	0.89640 (12)	0.6831 (3)	0.69114 (15)	0.0587 (4)	
H4A	0.9564	0.6187	0.7018	0.088*	
O5	1.25568 (15)	0.6306 (3)	0.62272 (16)	0.0699 (5)	

### Atomic displacement parameters (Å^2^)

**Table:**

	*U*^11^	*U*^22^	*U*^33^	*U*^12^	*U*^13^	*U*^23^
C4	0.0425 (9)	0.0284 (10)	0.0798 (14)	0.0016 (9)	0.0099 (9)	−0.0007 (10)
C1	0.0267 (8)	0.0497 (12)	0.0590 (11)	0.0022 (8)	0.0121 (7)	0.0057 (9)
C2	0.0395 (10)	0.0673 (16)	0.0785 (14)	0.0138 (11)	0.0234 (9)	−0.0065 (13)
C3	0.0552 (12)	0.0443 (13)	0.1029 (19)	0.0162 (12)	0.0243 (12)	−0.0044 (14)
C5	0.0392 (9)	0.0365 (11)	0.0678 (12)	0.0053 (8)	0.0020 (8)	0.0146 (9)
C6	0.0446 (9)	0.0423 (11)	0.0482 (9)	0.0010 (9)	0.0048 (7)	0.0152 (9)
C7	0.0378 (8)	0.0413 (10)	0.0334 (8)	0.0048 (8)	0.0042 (6)	0.0035 (7)
C8	0.0267 (7)	0.0312 (8)	0.0314 (7)	0.0029 (7)	0.0055 (5)	0.0007 (6)
C11	0.0291 (7)	0.0397 (10)	0.0333 (7)	0.0077 (7)	0.0071 (6)	0.0021 (7)
C12	0.0360 (8)	0.0408 (10)	0.0408 (8)	0.0094 (8)	0.0072 (6)	0.0056 (8)
C9	0.0277 (7)	0.0306 (9)	0.0363 (7)	0.0006 (7)	0.0029 (6)	0.0040 (7)
C10	0.0268 (7)	0.0444 (10)	0.0399 (8)	0.0005 (7)	0.0086 (6)	0.0033 (8)
C14	0.0522 (12)	0.100 (2)	0.0787 (16)	0.0113 (15)	−0.0105 (11)	0.0245 (18)
C13	0.0323 (9)	0.0586 (15)	0.0895 (16)	−0.0032 (10)	−0.0064 (9)	0.0044 (13)
C15	0.0285 (7)	0.0589 (12)	0.0347 (8)	0.0033 (8)	0.0054 (6)	0.0019 (8)
C16	0.0464 (9)	0.0478 (11)	0.0451 (9)	−0.0003 (9)	0.0186 (7)	−0.0042 (9)
C17	0.0723 (14)	0.0625 (16)	0.0556 (11)	−0.0063 (13)	0.0270 (10)	0.0036 (12)
C19	0.0363 (9)	0.0637 (15)	0.0590 (11)	−0.0096 (10)	0.0120 (8)	−0.0182 (11)
C18	0.0537 (11)	0.0563 (15)	0.0977 (17)	−0.0141 (12)	0.0234 (12)	−0.0159 (14)
N	0.0299 (6)	0.0480 (9)	0.0392 (7)	0.0003 (7)	0.0093 (5)	−0.0058 (7)
O1	0.0481 (7)	0.0642 (11)	0.0607 (8)	0.0229 (8)	0.0098 (6)	0.0236 (8)
O2	0.0364 (6)	0.0698 (11)	0.0645 (8)	0.0062 (8)	0.0226 (6)	0.0214 (8)
O3	0.0344 (6)	0.0383 (7)	0.0469 (6)	0.0060 (6)	0.0096 (5)	0.0122 (6)
O4	0.0453 (7)	0.0417 (8)	0.0877 (10)	−0.0077 (7)	0.0099 (7)	0.0103 (8)
O5	0.0682 (10)	0.0662 (12)	0.0845 (11)	−0.0174 (10)	0.0375 (9)	0.0003 (10)

### Geometric parameters (Å, °)

**Table:**

C4—C5	1.320 (3)	C9—C10	1.495 (2)
C4—C3	1.509 (3)	C9—H9	0.9800
C4—H4	0.9300	C10—O2	1.444 (2)
C1—O2	1.452 (3)	C10—H10	0.9800
C1—C10	1.466 (2)	C14—H14A	0.9600
C1—C2	1.503 (4)	C14—H14B	0.9600
C1—C13	1.509 (3)	C14—H14C	0.9600
C2—C3	1.533 (4)	C13—H13A	0.9600
C2—H2A	0.9700	C13—H13B	0.9600
C2—H2B	0.9700	C13—H13C	0.9600
C3—H3A	0.9700	C15—N	1.473 (3)
C3—H3B	0.9700	C15—H15A	0.9700
C5—C14	1.519 (3)	C15—H15B	0.9700
C5—C6	1.520 (3)	C16—N	1.465 (2)
C6—O4	1.418 (3)	C16—C17	1.509 (3)
C6—C7	1.537 (3)	C16—H16A	0.9700
C6—H6	0.9800	C16—H16B	0.9700
C7—C8	1.540 (2)	C17—O5	1.410 (3)
C7—H7A	0.9700	C17—H17A	0.9700
C7—H7B	0.9700	C17—H17B	0.9700
C8—C11	1.538 (2)	C19—N	1.467 (3)
C8—C9	1.540 (2)	C19—C18	1.496 (4)
C8—H8	0.9800	C19—H19A	0.9700
C11—C12	1.513 (2)	C19—H19B	0.9700
C11—C15	1.518 (2)	C18—O5	1.433 (3)
C11—H11	0.9800	C18—H18A	0.9700
C12—O1	1.198 (2)	C18—H18B	0.9700
C12—O3	1.342 (2)	O4—H4A	0.8200
C9—O3	1.459 (2)		
			
C5—C4—C3	128.1 (2)	C8—C9—H9	110.7
C5—C4—H4	116.0	O2—C10—C1	59.83 (12)
C3—C4—H4	116.0	O2—C10—C9	119.60 (17)
O2—C1—C10	59.33 (11)	C1—C10—C9	125.82 (16)
O2—C1—C2	116.72 (19)	O2—C10—H10	113.6
C10—C1—C2	115.79 (19)	C1—C10—H10	113.6
O2—C1—C13	112.9 (2)	C9—C10—H10	113.6
C10—C1—C13	122.61 (19)	C5—C14—H14A	109.5
C2—C1—C13	116.52 (19)	C5—C14—H14B	109.5
C1—C2—C3	112.15 (19)	H14A—C14—H14B	109.5
C1—C2—H2A	109.2	C5—C14—H14C	109.5
C3—C2—H2A	109.2	H14A—C14—H14C	109.5
C1—C2—H2B	109.2	H14B—C14—H14C	109.5
C3—C2—H2B	109.2	C1—C13—H13A	109.5
H2A—C2—H2B	107.9	C1—C13—H13B	109.5
C4—C3—C2	111.12 (19)	H13A—C13—H13B	109.5
C4—C3—H3A	109.4	C1—C13—H13C	109.5
C2—C3—H3A	109.4	H13A—C13—H13C	109.5
C4—C3—H3B	109.4	H13B—C13—H13C	109.5
C2—C3—H3B	109.4	N—C15—C11	113.24 (13)
H3A—C3—H3B	108.0	N—C15—H15A	108.9
C4—C5—C14	125.7 (2)	C11—C15—H15A	108.9
C4—C5—C6	121.92 (18)	N—C15—H15B	108.9
C14—C5—C6	112.3 (2)	C11—C15—H15B	108.9
O4—C6—C5	111.44 (19)	H15A—C15—H15B	107.7
O4—C6—C7	111.71 (16)	N—C16—C17	109.68 (19)
C5—C6—C7	111.18 (17)	N—C16—H16A	109.7
O4—C6—H6	107.4	C17—C16—H16A	109.7
C5—C6—H6	107.4	N—C16—H16B	109.7
C7—C6—H6	107.4	C17—C16—H16B	109.7
C6—C7—C8	115.55 (16)	H16A—C16—H16B	108.2
C6—C7—H7A	108.4	O5—C17—C16	112.00 (19)
C8—C7—H7A	108.4	O5—C17—H17A	109.2
C6—C7—H7B	108.4	C16—C17—H17A	109.2
C8—C7—H7B	108.4	O5—C17—H17B	109.2
H7A—C7—H7B	107.5	C16—C17—H17B	109.2
C11—C8—C7	113.64 (13)	H17A—C17—H17B	107.9
C11—C8—C9	103.05 (13)	N—C19—C18	110.83 (18)
C7—C8—C9	116.19 (13)	N—C19—H19A	109.5
C11—C8—H8	107.9	C18—C19—H19A	109.5
C7—C8—H8	107.9	N—C19—H19B	109.5
C9—C8—H8	107.9	C18—C19—H19B	109.5
C12—C11—C15	109.82 (13)	H19A—C19—H19B	108.1
C12—C11—C8	104.29 (12)	O5—C18—C19	111.8 (2)
C15—C11—C8	114.26 (16)	O5—C18—H18A	109.3
C12—C11—H11	109.4	C19—C18—H18A	109.3
C15—C11—H11	109.4	O5—C18—H18B	109.3
C8—C11—H11	109.4	C19—C18—H18B	109.3
O1—C12—O3	121.22 (17)	H18A—C18—H18B	107.9
O1—C12—C11	127.88 (16)	C16—N—C19	108.64 (14)
O3—C12—C11	110.89 (14)	C16—N—C15	111.03 (17)
O3—C9—C10	107.07 (13)	C19—N—C15	109.89 (14)
O3—C9—C8	106.16 (11)	C10—O2—C1	60.83 (11)
C10—C9—C8	111.28 (15)	C12—O3—C9	111.20 (13)
O3—C9—H9	110.7	C6—O4—H4A	109.5
C10—C9—H9	110.7	C17—O5—C18	109.61 (17)

### Hydrogen-bond geometry (Å, °)

**Table:**

*D*—H···*A*	*D*—H	H···*A*	*D*···*A*	*D*—H···*A*
O4—H4A···N	0.82	2.24	3.051 (2)	172
C2—H2B···O2^i^	0.97	2.51	3.324 (3)	142
C10—H10···O1^ii^	0.98	2.47	3.270 (2)	138

Symmetry codes:  (i) −*x*+1, *y*+1/2, −*z*+2; (ii) −*x*+2, *y*+1/2, −*z*+2.
